# The Dynamics of DNA Methylation Covariation Patterns in Carcinogenesis

**DOI:** 10.1371/journal.pcbi.1003709

**Published:** 2014-07-10

**Authors:** Andrew E. Teschendorff, Xiaoping Liu, Helena Caren, Steve M. Pollard, Stephan Beck, Martin Widschwendter, Luonan Chen

**Affiliations:** 1 CAS-MPG Partner Institute for Computational Biology, Chinese Academy of Sciences, Shanghai Institute for Biological Sciences, Shanghai, China; 2 Statistical Genomics Group, UCL Cancer Institute, University College London, London, United Kingdom; 3 Key Laboratory of Systems Biology, SIBS-Nordisk Translational Research Centre for PreDiabetes, Shanghai Institute for Biological Sciences, Chinese Academy of Sciences, Shanghai, China; 4 Sahlgrenska Cancer Center, Department of Pathology, University of Gothenburg, Gothenburg, Sweden; 5 Department of Cancer Biology, UCL Cancer Institute, University College London, London, United Kingdom; 6 Medical Genomics Group, UCL Cancer Institute, University College London, London, United Kingdom; 7 Department of Women's Cancer, UCL EGA Institute for Women's Health, University College London, London, United Kingdom; Ottawa University, Canada

## Abstract

Recently it has been observed that cancer tissue is characterised by an increased variability in DNA methylation patterns. However, how the correlative patterns in genome-wide DNA methylation change during the carcinogenic progress has not yet been explored. Here we study genome-wide inter-CpG correlations in DNA methylation, in addition to single site variability, during cervical carcinogenesis. We demonstrate how the study of changes in DNA methylation covariation patterns across normal, intra-epithelial neoplasia and invasive cancer allows the identification of CpG sites that indicate the risk of neoplastic transformation in stages prior to neoplasia. Importantly, we show that the covariation in DNA methylation at these risk CpG loci is maximal immediately prior to the onset of cancer, supporting the view that high epigenetic diversity in normal cells increases the risk of cancer. Consistent with this, we observe that invasive cancers exhibit increased covariation in DNA methylation at the risk CpG sites relative to normal tissue, but lower levels relative to pre-cancerous lesions. We further show that the identified risk CpG sites undergo preferential DNA methylation changes in relation to human papilloma virus infection and age. Results are validated in independent data including prospectively collected samples prior to neoplastic transformation. Our data are consistent with a phase transition model of carcinogenesis, in which epigenetic diversity is maximal prior to the onset of cancer. The model and algorithm proposed here may allow, in future, network biomarkers predicting the risk of neoplastic transformation to be identified.

## Author Summary

DNA methylation is a covalent modification of DNA which can regulate how active genes are. DNA methylation is altered at many genomic loci in cancer cells, leading to widespread functional disruption. Importantly, DNA methylation alterations across the genome are seen even in early carcinogenesis. Although the pattern of DNA methylation change during carcinogenesis has been studied at individual genomic loci, no study has yet analysed how these patterns change at a systems-level, specifically how do DNA methylation patterns at pairs of genomic sites change during disease progression. Doing so can shed light on how the epigenetic diversity of cell populations changes during the carcinogenic process. This study performs a systems-level analysis of the dynamic changes in DNA methylation correlation pattern during cervical carcinogenesis, demonstrating that epigenetic diversity is maximal just prior to the onset of cancer. Importantly, this supports the view that the risk of cancer development is closely related to an increase in epigenetic diversity in apparently healthy cells. In addition, the study provides a computational algorithm which successfully identifies the altered genomic sites confering the risk of cervical cancer.

## Introduction

Cancer exhibits widespread DNA methylation (DNAm) changes compared to normal tissue [1]–[7]. As demonstrated by a number of studies, genomic sites undergoing DNA methylation changes in invasive cancers are already seen to accumulate changes in normal tissue as a function of age [8]–[11]. All these observations have been derived by analysing the changes in mean DNA methylation levels at specific genomic sites. More recently, studies have begun to explore and demonstrate the importance of DNA methylation variability in cancer and other complex diseases [11]–[15]. As shown by Hansen et al, cancer tissue is characterised not only by changes in mean levels of DNA methylation, but importantly also by increases in DNA methylation variability [12]. This increased variability is seen at specific genomic regions, both spatially within the region and the given sample, as well as across samples. This latter inter-sample variability suggests that the variation may be due to increased stochasticity [12], [13], [16]. The importance of considering DNA methylation variability across samples was subsequently demonstrated in the context of a prospective study in cervical cancer: it was shown that cytologically normal samples, collected three years in advance of neoplastic transformation, exhibited increased levels of inter-sample DNA methylation variation compared to age-matched normal samples which did not progress to neoplasia [13]. Furthermore, by developing a novel statistical algorithm called EVORA (Epigenetic Variable Outlier for Risk Prediction Analysis), it was shown that differential DNAm variability could identify risk markers more robustly than statistical measures based on differences in mean DNAm levels [17]. This result was attributed to the risk CpG markers exhibiting outlier DNAm profiles, in which only a small subgroup of samples exhibit aberrant DNA methylation. This again supports the view that DNAm changes arising before the onset of cancer are, in part, heterogeneous and stochastic [13], [17].

From a statistical perspective, all studies so far have only explored the dynamic changes in the mean and variance of DNA methylation during carcinogenesis. As yet, no study has fully explored the changes in DNA methylation correlations that accompany the neoplastic process. We refer to the study of correlations between molecular features and their variance as “covariation”.

Here, we decided to explore the dynamic changes in genome-wide DNA methylation covariation patterns that happen during the carcinogenic process. We hypothesized that dynamic changes in the DNAm correlation patterns could shed further light on the carcinogenic process itself. Specifically, we sought to determine if the covariation in DNAm progresses in a linear fashion with a maximum in the invasive cancer stage (as hinted by a previous study [12]), or if instead, the covariation exhibits a non-linear dynamics. By using genome-wide DNA methylation data from the main stages in cervical carcinogenesis, we here demonstrate the existence of CpG loci whose covariation in DNAm progresses in a non-linear fashion, exhibiting a maximum in a disease stage *prior to the onset of cancer*. This non-linear dynamics is reminiscent of an underlying phase transition model of disease progression [18], which we adapt to the epigenetics context and then validate in independent cohorts, including prospectively collected samples. Importantly, we demonstrate how the proposed model allows identification of a network biomarker able to predict the risk of neoplastic transformation three years in advance of transformation.

## Results

### The Dynamical Network Biomarker (DNB) algorithm

We decided to explore the dynamic changes in DNA methylation covariation patterns during carcinogenesis in the context of cervical cancer, since for this cancer the cell of origin is known and is easily accessible in advance of neoplastic transformation as part of large routine screening programs and clinical trials (e.g. the ARTISTIC trial) [13], [19]. However, even for cervical cancer, two major obstacles emerge. First, the analysis of dynamic changes requires extensive time-course data from the same individual prior to and subsequent to disease diagnosis, yet such extensive time-course data is not available. Second, measuring the methylation state of say 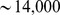 gene promoters translates into the need to analyze well over 9 million pairwise correlations, which is a computationally demanding task.

To overcome these challenges, we adapted a physical model of disease progression, first proposed by Chen et al [18], to the DNA methylation context (**Materials and Methods**). Briefly, the model assumes that disease progresses through a series of physical phase transitions and that those molecular features (i.e. in our context these are CpGs) exhibiting a phase transition type behaviour may be particular important for the progression of that specific disease. By phase transition behaviour we mean an abrupt increase and subsequent decrease in the covariation strength of a set of CpGs which coincides with the transition between two successive disease stages. Importantly however the model is otherwise relatively assumption-free, and provides a numerical prescription, called the Dynamical Network Biomarker (DNB) algorithm, to identify sets of CpGs, called CpG-modules, which may exhibit phase-transition like behaviours between progressive disease stages. A module that does exhibit a non-linear phase-transition like pattern is called a Dynamical Network Biomarker (DNB) and here we wanted to assess the possibility that such a module could be used to indicate the risk of disease progression.

**Figure 1:**
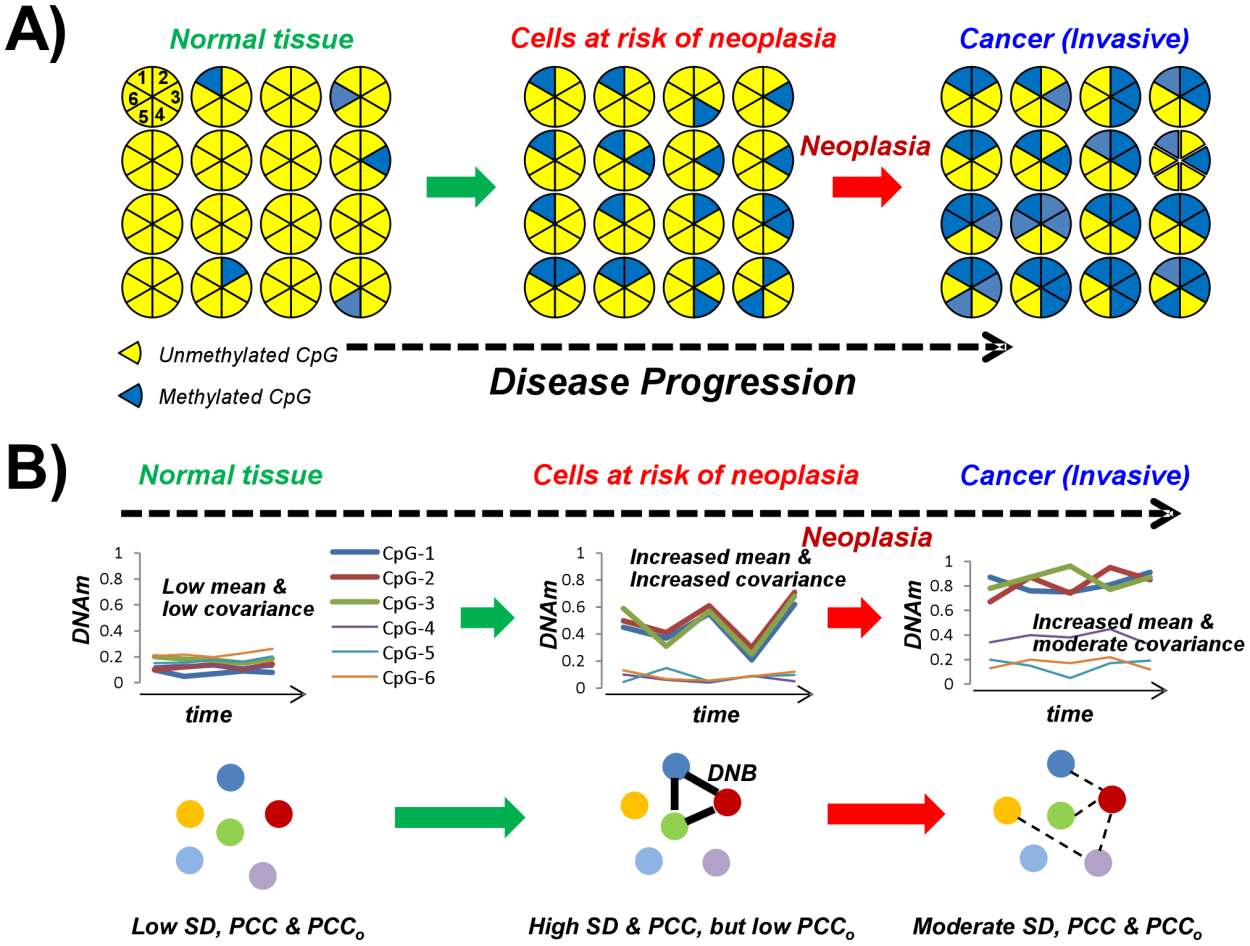
The dynamics of DNA methylation in carcinogenesis. **A**) Progressive changes in DNA methylation are shown for a number of cells and for three disease stages, as shown. For each cell we only depict 6 CpG sites, which are assumed to map to high-CpG dense promoters and thus most start out as unmethylated (yellow colour). With time, some of these CpGs acquire methylation (blue) and once aquired these are relatively stable marks. However, at the cellular population level, the hypothesis is that DNA methylation patterns become least predictable, i.e become most stochastic and diverse, in stages just prior to the onset of neoplasia. Measured in time, covariances in DNA methylation will be maximal in this high risk stage because no dominant subclone exists. Consistent with observations, CpG sites become more homogeneously methylated (more predictable) once the cancer has developed. **B**) Upper panel: Hypothesized pattern of variation of the average DNA methylation as measured over a population of cells in each disease stage. Depicted are the patterns for 6 hypothetical CpGs. Observe how the variability in DNA methylation for certain CpGs would be maximal in the high-risk stage, due to temporal variations in the dominating subclone population. Lower panel: Relevance network representation of the correlation strengths with disease stage with thick edges representing strong correlations. CpGs exhibiting maximal variation (SD) and covariation (PCC), but low correlation to other CpGs (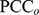), at the transition point to neoplasia define a Dynamic Network Biomarker (DNB).

The DNB algorithm circumvents the problem of temporal data by approximating the unobserved temporal correlations in DNA methylation between CpGs within a sample by non-temporal correlations estimated over independent samples, i.e. from different individuals but crucially all representing the same disease stage [18] (**Materials and Methods**). This approximation assumes that independently collected samples within a disease stage represent slightly different time points of disease progression of one representative individual within that same disease stage. To justify the approximation, we conducted a detailed simulation study, generating temporal DNA methylation data for a number of CpGs and individuals and then subsampling one time point for each individual (**Materials and Methods**). DNAm profiles of CpGs making up artificial DNBs were generated according to a phase transition type model in which the variation and co-variation in DNAm increases and becomes maximal just before the transition point (e.g. the onset of neoplasia) is reached (**Materials and Methods**). We note that such a model is consistent with the view that epigenetic plasticity may be highest in stages prior to the onset of neoplastic transformation (**Fig. 1**). As observed across a number of different individuals this means that epigenetic profiles may be least predictable (i.e appear most stochastic) in the stage just prior to neoplasia and cancer. Applying the DNB algorithm to the simulated data revealed sensitivity values around 0.5, indicating that the non-temporal approximation may be able to capture DNBs in real data (**Fig. S1 in Text S1**). Thus, based on this simulated data, it is therefore possible to apply the DNB algorithm on real data using a coarse-grained time variable with as many time points as there are disease stages.

The DNB algorithm also circumvents the problem posed by the high-dimensional correlation space (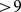 million correlations), since it uses a semi-supervised clustering approach to perform dimensional reduction, whereby the salient dynamical changes are captured by a relatively small number of “gene-modules” (in our context “CpG-modules”) (**Materials and Methods**). Thus, for each data set representing a given disease stage, the DNB algorithm infers a number of CpG-modules (**Materials and Methods**). For each of these inferred CpG-modules and in each disease stage, one then computes a “relevance score” which measures the amount of DNAm variation and the strength of DNAm covariation of the CpG-module members. Specifically, the score for a given module 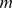 in disease stage 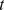 is estimated by the formula
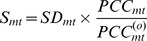
(1)where 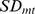 is the average standard deviation of the DNA methylation profiles of the CpGs making up the module as assessed over samples within disease stage 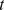, 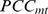 denotes their average pairwise Pearson correlation coefficient, and 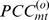 denotes the average Pearson correlation between the module features and their complement, i.e. all other non-module CpGs (**Materials and Methods**). As shown by Chen et al, this heuristic score can be motivated solely on theoretical principles [18] (see also **Materials and Methods**). However, as we argue next, it can also be motivated biologically (**Fig. 1**). Specifically, we seek groups of CpGs with large co-variability in specific disease stages, which means high variability and absolute pairwise correlations. Thus, the relevance score should be proportional to 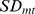, since this represents the average variability in DNAm of these CpGs in a given disease stage 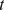. Similarly, the score should be proportional to the ratio 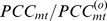, since this measures the coherence of the module CpG's DNAm correlations relative to the rest of the assayed CpGs (**Fig. 1A–B**). Thus, for a given fixed module 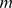, studying how the score 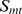 changes as a function of disease stage 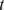 will inform us about the dynamic changes in DNAm covariation patterns that the module CpGs/genes incur during carcinogenesis. By construction, this score is likely to be maximal for the disease stage in which the module was inferred. However, for the same module, the score it obtains in other disease stages will provide highly non-trivial information about the underlying dynamics and biological relevance of its member CpGs/genes during carcinogenesis. We can further use independent data from similar or other disease stages to validate the score predictions of the modules derived from the training data.

### The dynamics of DNA methylation covariation patterns in cervical carcinogenesis

**Figure 2:**
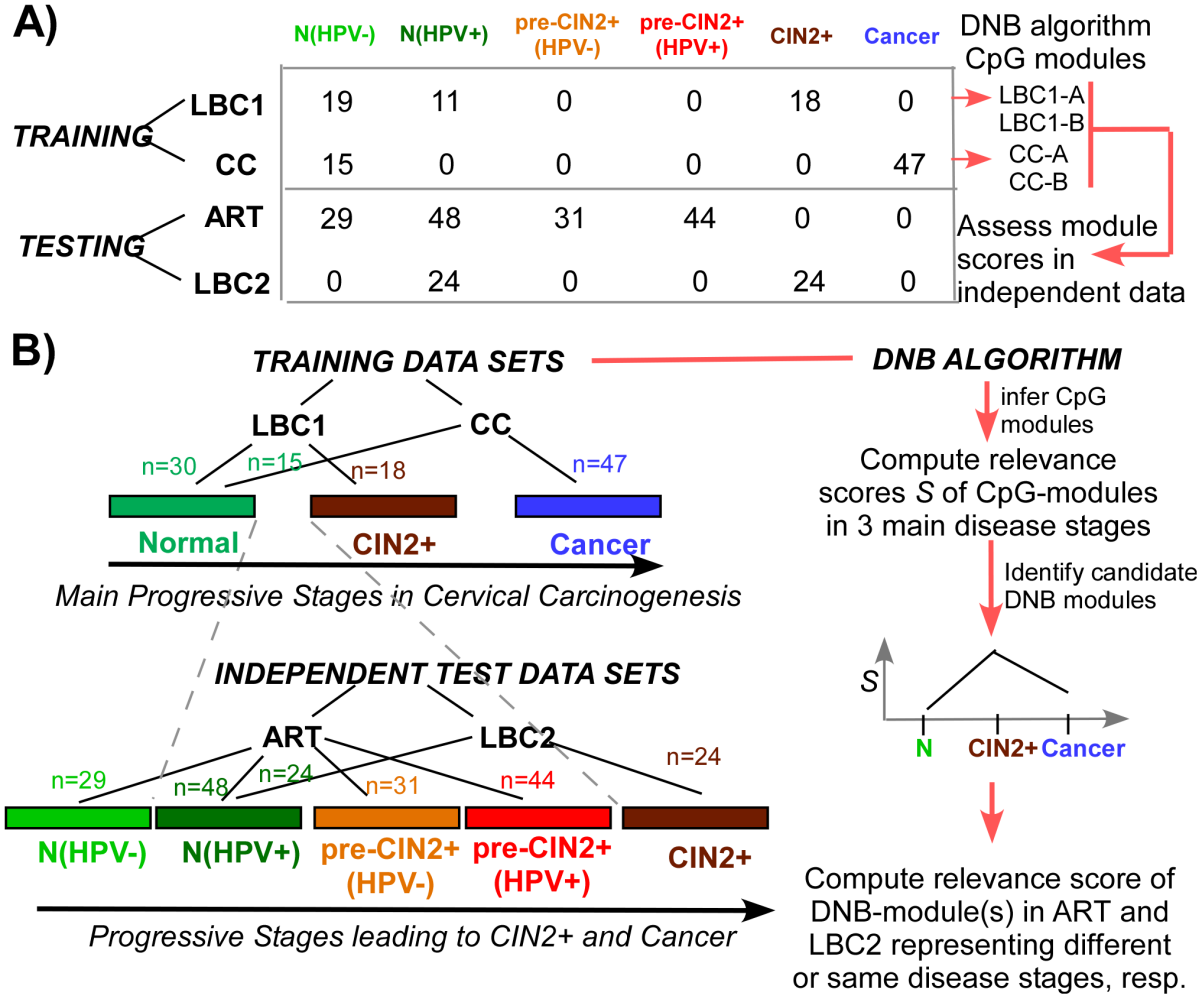
Datasets and analysis strategy used. **A**) Distribution of samples according to dataset (LBC1, CC, ART, LBC2) and disease stage in cervical carcinogenesis. Datasets LBC1 and CC were used for training, i.e. the DNB algorithm was applied to these sets only to infer candidate DNB modules. Datasets ART and LBC2 were used to test the predictions of the module scores obtained in the training data. **B**) The overall analysis strategy was to use LBC1 and CC as training sets, to infer candidate DNB modules across the 3 main stages of cervical carcinogenesis: normal, CIN2+ (cervical intraepithelial neoplasia of grade 2 or higher) and invasive cervical cancer, as shown. After computation of the relevance scores, measuring the strength of covariation in DNAm, of the inferred modules, we identified a candidate DNB(s) as the one exhibiting a maximum in the score in a stage (CIN2+) prior to invasive cancer. Finally, for this DNB module, we compute its score in independent data sets profiling samples from a previously considered disease stage (i.e. LBC2 for CIN2+) or from other intermediate disease stages (e.g. ART for normal HPV−, normal HPV+, precursor CIN2+ HPV− and precursor CIN2+ HPV+ cells). Prediction is that the scores in the CIN2+ LBC2 samples should agree with those of the CIN2+ LBC1 samples, and that the score values in disease stages N(HPV+), pre-CIN2+(HPV−) and pre-CIN2+(HPV+) should be intermediate between N(HPV−) and CIN2+.

In order to test the DNB algorithm in the DNA methylation context, we collected DNA methylation data from six progressive stages in cervical carcinogenesis, all generated using the same Illumina Infinium 27k platform [20], which measures DNA methylation at over 27,000 CpG sites (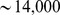 gene promoters). Because infection by the human papilloma virus (HPV) is a necessary, but not sufficient, factor for cervical cancer [19], normal samples were stratified according to HPV status (if known). Thus, the six disease stages were HPV− normal cells, HPV+ normal cells, HPV− normal cells which become a cervical intraepithelial neoplasia of grade 2 or higher (CIN2+) three years later (“CIN2+ precursor cells”), HPV+ CIN2+ precursor cells, CIN2+ cells and invasive cervical cancer (**Fig. 2A**). The DNA methylation data was drawn from a total of four different studies, abbreviated as ART, LBC1, LBC2 and CC (**Fig. 2A**). The ART data set consists of 152 samples, encompassing the first four disease stages. To clarify, these 152 samples are all cytologically normal, yet 75 of these became CIN2+ after 3 years and are thus denoted “precursor CIN2+”. Data sets LBC1 and LBC2 are similar in that they both contain CIN2+ samples, yet only LBC1 contains normal HPV− samples, whilst the normal samples in LBC2 were HPV+ (**Fig. 2A**). Finally, data set CC contains invasive cervical cancers in addition to normal cervical samples (presumed HPV−).

To infer modules of CpGs and to investigate their broad dynamic changes in DNAm covariation, we first focused on samples from only the 3 main stages: HPV− normal cells, CIN2+ cells, and invasive cancer. These samples were drawn from data sets LBC1 and CC, which constitute our training data (**Figs. 2A–B**). Application of the DNB algorithm to these two data sets resulted in four distinct CpG modules called LBC1-A, LBC1-B, CC-A and CC-B, the terminology reflecting the data set which they were derived from (**Fig. S2 in Text S1**). To clarify, we note that inference of CpG modules via the DNB algorithm requires samples which represent a normal reference (**Materials and Methods**). Thus, in deriving LBC1-A and LBC1-B we used as reference the normal samples of data set LBC1, whereas for CC-A and CC-B we used the normal samples of data set CC. This strategy ensures that results are not confounded by study-specific effects.

**Figure 3:**
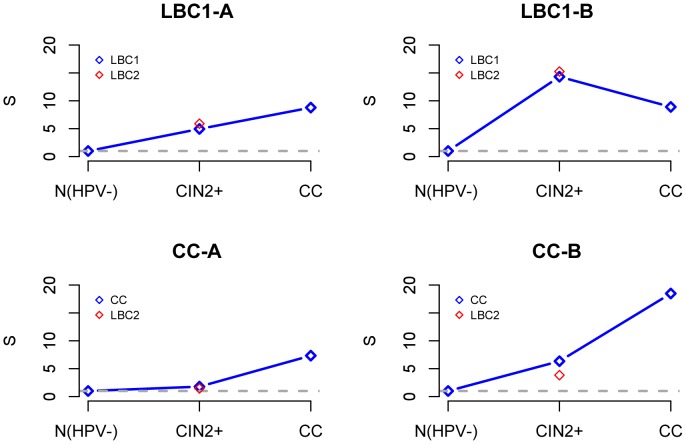
Broad dynamic changes of inferred CpG-modules. The changes in the relevance score as a function of main disease stage (as indicated) for each of the inferred modules. LBC1-A and LBC1-B were derived from comparing CIN2+ to normals (HPV− normals) in set LBC1, while modules CC-A and CC-B were derived by comparing cervical cancers (CC) to HPV− normal cervical tissue in data set CC. The stages shown are N(HPV−), CIN2+, and cervical cancer (CC). In red, we show the relevance score attained by these same modules in an independent test data set consisting of CIN2+ samples (LBC2 set).

Estimation of the relevance (i.e. covariation strength) scores of these four modules in each of the 3 main disease stages revealed highly distinctive dynamical behaviour, with LBC1-B and CC-A modules exhibiting sharp increases specific to their disease stage (**Fig. 3**). Curiously, module LBC1-B, in contrast to LBC1-A, exhibited a clear maximum in the CIN2+ disease stage, i.e prior to invasive cancer. To demonstrate that this is not an artefact of the module construction and score estimation procedure, we evaluated the relevance score of this same module in an independent data set encompassing CIN2+ samples (LBC2 set, **Fig. 2**). The score value attained by the LBC1-B module in this independent set was highly concordant with that in the training data, a result which was also true for the other inferred modules (**Fig. 3**). Thus, the sharp maximum exhibited by the LBC1-B module in the CIN2+ disease stage is a biological feature of the CpG sites making up the module and not the result of overfitting.

### Non-linear dynamics of DNAm covariation patterns signals the transition to neoplasia

**Figure 4:**
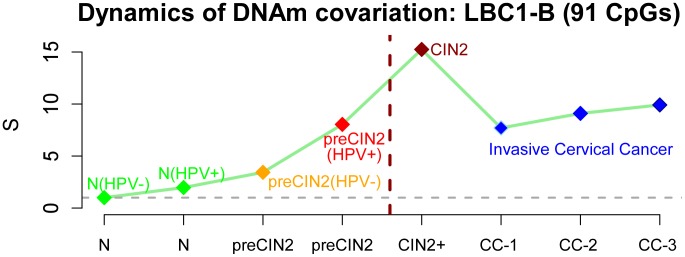
Dynamical Network Biomarker (DNB) in cervical carcinogenesis. The changes in the relevance score of the LBC1-B CpG module, termed a DNB, as a function of disease stage: the stages shown are N(HPV−), N(HPV+), preCIN2(HPV−), preCIN2(HPV+), CIN2+, CC stages 1,2 and 3. Note that the samples from stages N(HPV+), preCIN2(HPV−) and preCIN2(HPV+) were drawn from completely independent test sets and that these samples exhibit relevance scores which are intermediate between N(HPV−) and CIN2+, in line with their disease stage. Darkred dashed line indicates hypothetical switching point in the transition from cytologically normal cells at risk of CIN2+ to CIN2+. (Abbrev: N = Normal, preCIN2+: precursor CIN2+ cells, CIN2+ = cervical intraepithelial neoplasia of grade 2 or higher, CC = cervical cancer).

The non-linear dynamics with the sharp maximum exhibited by the LBC1-B module is reminiscent of an underlying phase transition model, and thus we posited that these specific CpGs could be specially important for cervical carcinogenesis. Indeed, we posited that the relevance score of these sites may already show increases in disease stages that precede CIN2+ and could thus be used for risk prediction. To test this, we computed the relevance score of the LBC1-B module CpGs in independent samples representing HPV+ normal cells, as well as HPV− and HPV+ precsursor CIN2+ cells. These precursor samples represent cytologically normal samples at measurement, but which 3 years later became CIN2+ and were drawn from the ART data set (**Fig. 2**, [13]). Remarkably, the relevance score of the LBC1-B module estimated in these independent samples representing these intermediate disease stages were also intermediate, lying in between those of normal HPV− cells and CIN2+ samples (**Fig. 4**). In fact, the relevance score of the LBC1-B module exhibited a gradual monotonic increase from normal HPV− cells to normal HPV+ cells, to precursor HPV− and HPV+ CIN2+ cells and finally to the CIN2+ stage (**Fig. 4**). In order to further test the robustness of the data, we also recomputed the relevance score of this module for invasive cervical cancers stratified according to their stages, and these were never higher than for the CIN2+ disease stage (**Fig. 4**). We also computed the relevance scores of the other 3 modules in these independent samples, and interestingly, none of the other 3 modules exhibited a maximum prior to the onset of invasive cancer (**Fig. S3 in Text S1**). Thus, this highlights the distinctive nature of the LBC1-B module.

**Figure 5:**
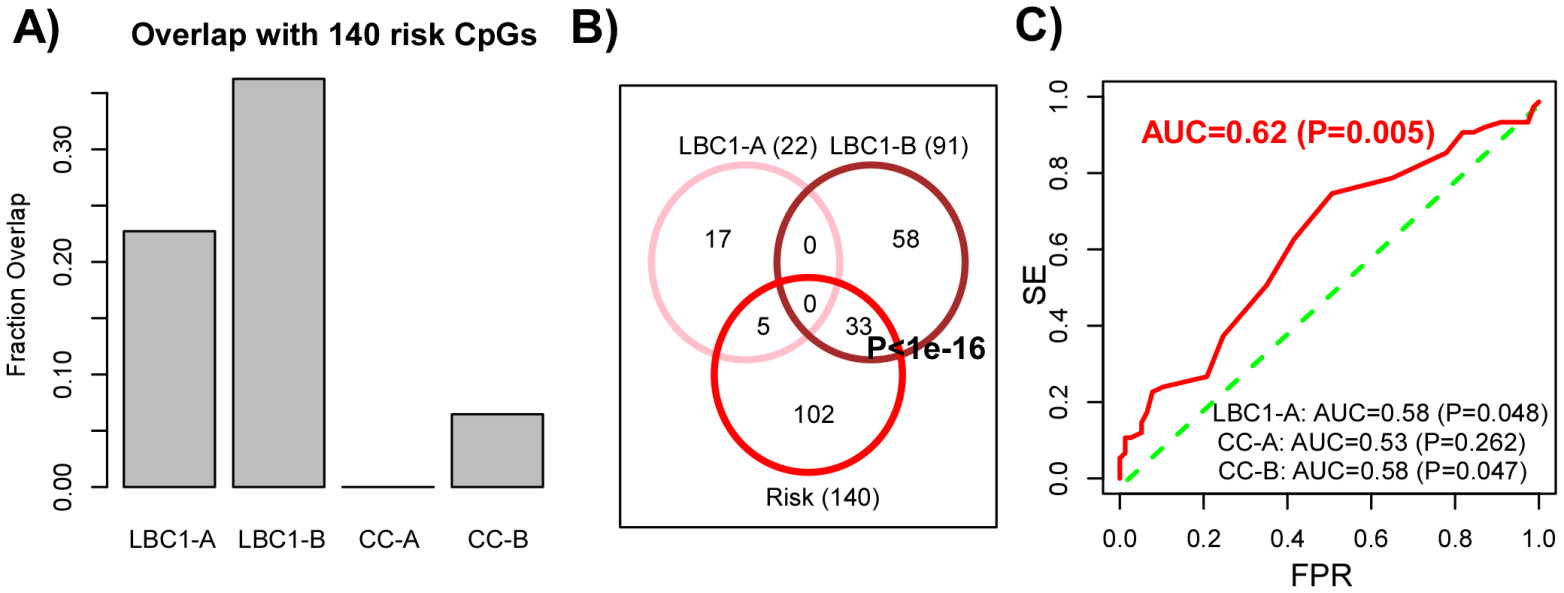
DNB module predicts risk of neoplastic transformation. **A**) Fractional overlaps of the four inferred modules with the 140 risk CpG set identified using EVORA [13] in the ART cohort. **B**) Statistically significant overlap of the 91 DNB module CpGs with the 140 risk CpGs. Note that by construction, none of the CpG markers in module LBC1-A overlap with those in the DNB (LBC1-B). **C**) ROC AUC analysis for the average methylation risk score for the DNB (LBC1-B) module. We provide the AUC and associated P-values of significance for LBC1-B (in red), and for the other 3 modules (in black).

To test if the LBC1-B module can predict the risk of neoplastic transformation, we first asked if the CpGs making up the LBC1-B module overlapped with the 140 risk-associated CpGs previously derived using the EVORA algorithm [13]. We observed that the LBC1-B module, consisting of 91 CpG markers, exhibited a strong overlap (33/91, 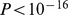, **Fig. 5A–B**) with the 140 risk CpGs reported in [13]. This is remarkable given that the LBC1-B module and the 140 risk CpGs were derived from independent data sets and different disease stages (LBC1 and ART, respectively). Not surprisingly, the LBC1-B markers were highly enriched for PolyComb Group Target genes (PCGTs) [21] (Fisher test, Benjamini Hochberg adjusted 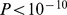), in line with the observed strong PCGT enrichment of the 140 risk CpG sites [13]. Importantly none of the other modules exhibited an overlap with the 140 risk CpG sites as strong as our candidate DNB (**Fig. 5A**).

To formally demonstrate that the inferred LBC1-B module can predict the risk of neoplasia we computed an average methylation risk score over the 91 CpG markers for each sample in the ART set (see **Fig. 2A**, [13]). This risk score was predictive of prospective CIN2+ status with an AUC = 0.62 (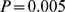, **Fig. 5C**), similar to the AUC of the 140 risk CpGs reported in [13] and higher than that of the other inferred modules (**Fig. 5C**). Thus, LBC1-B is a candidate dynamical network biomarker (DNB), signaling the transition to a neoplastic state.

### Increased covariance of risk CpG sites prior to CIN2+ is followed by homogenization in invasive cancers

**Figure 6:**
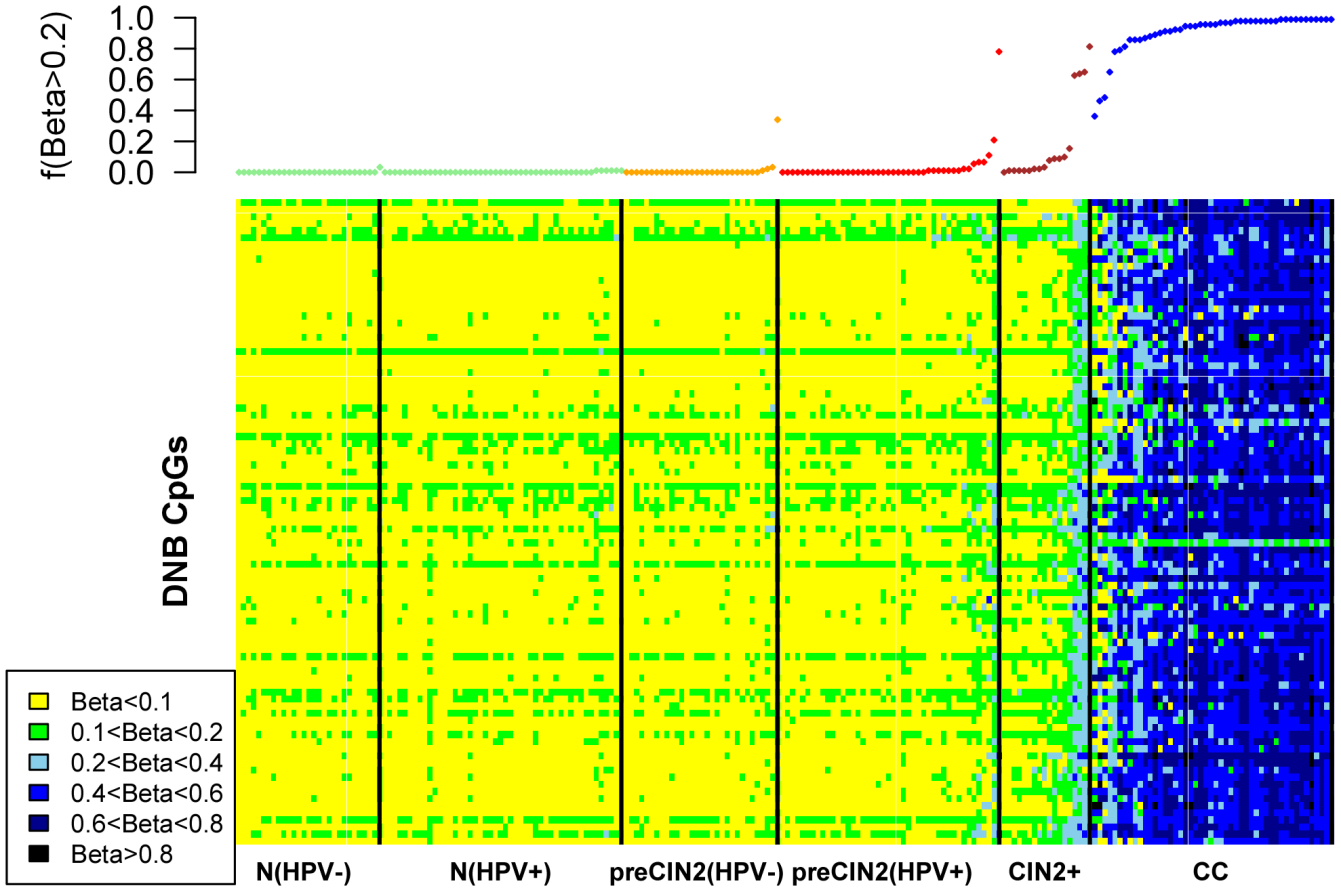
DNA methylation changes of DNB markers during cervical carcinogenesis. Heatmap depicts the DNAm levels of the 91 CpGs making up the DNB module (LBC1-B) across six stagtes in cervical carcinogenesis, as indicated. In each disease stage, samples have been ordered according to the average fraction of the 91 CpGs that exhibit a methylation beta-value larger than 0.2 (see upper panel).

That the relevance score of the LBC1-B/DNB module reaches a maximum in the CIN2+ stage, with a subsequent decline observed in invasive cancer indicates that for these particular CpG sites there is a reduction in the DNAm covariances as evaluated across the cancers. To understand why DNAm covariances in cancer may be reduced, we generated heatmaps of DNA methylation for the 91 DNB CpGs across all major disease stages ranking samples within each disease stage according to the fraction of methylated sites (using a relaxed 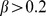 threshold) (**Fig. 6**). This fractional methylation score exhibited a striking bi-modality within the CIN2+ disease stage, with some CIN2+ samples exhibiting high methylation fractions and others exhibiting much lower levels (**Fig. 6**). Remarkably, almost all 91 CpGs acquired extensive methylation in the great majority of invasive cancers. Thus, this clearly demonstrates that CpG sites already undergoing changes before morphological transformation, continue to undergo further DNAm modification in CIN2+ and cancer cells. The observation that samples are particularly diverse and bi-modal in the CIN2+ stage suggests that epigenetic diversity may be highest in this disease stage, with those CIN2+ samples exhibiting the methylator phenotype (i.e. a high methylation fraction) being on course to becoming invasive cancers.

### Risk CpGs are specially associated with HPV status and age

Many of the risk CpGs making up the DNB module exhibit significant methylation increases in CIN2+ cells, since they were selected by comparing DNAm levels between CIN2+ and normal cells. Based on the observed gradual increase in the relevance score of the DNB from normal HPV− to normal HPV+, to precursor CIN2+ HPV− and subsequently to precursor CIN2+ HPV+ cells, we argued that the CpGs making up the DNB module would also be associated with HPV status. Averaging the methylation values over the 91 DNB CpGs for each sample in the ART dataset (**Fig. 2A**) confirmed a significant increase in the HPV+ precursor CIN2+ cells compared to their HPV− counterparts (**Fig. S4 in Text S1**). To further confirm this association with HPV status, we investigated the DNA methylation profiles of these 91 CpGs in head & neck cancer [22], another cancer for which HPV is a risk factor: we observed that these CpGs were overwhelmingly hypermethylated in HPV+ head & neck cancers compared to their HPV− counterparts (**Fig. S5 in Text S1**). We also asked if the average methylation over the 91 CpGs would correlate with age, another risk factor for CIN2+. Interestingly, this was indeed the case and it did so independently of HPV status and prospective CIN2+ status (**Fig. S4 in Text S1**). To further assess the biological significance of these 91 CpGs, we randomly selected another 91 CpGs from the same dataset, and recomputed the association between average methylation and HPV status or age. In only 2 of 10,000 random selections of 91 CpGs, did we observe discriminatory P-values as extreme as the ones for the actual 91 LBC1-B CpGs (**Fig. S4 in Text S1**, FDR 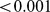 for both age and HPV status). Thus, this shows that these particular CpG sites undergo preferential methylation increases in normal tissue as a function of age and in pre-neoplastic lesions as a function of current HPV status.

## Discussion

To the best of our knowledge, this is the first study to analyse patterns of DNAm covariation during carcinogenesis. We used cervical cancer as our model since for this cancer it is possible to acquire relatively large numbers of samples prior to neoplastic transformation, thus allowing the stages prior to neoplasia and cancer to be assessed. From a computational perspective, analysing pairwise correlations in DNAm of ∼27,000 CpGs is technically very demanding, due to the need to analyse millions of pairwise correlations. To circumvent this problem, we used a statistical algorithm, grounded on physical principles [18], [23], to allow us to identify CpG modules exhibiting interesting dynamical changes in DNAm covariation patterns.

By applying this algorithm to only three disease stages of cervical carcinogenesis (normal, CIN2+, cancer), we identified one CpG module, called a dynamical network biomarker (DNB), whose covariation exhibited a non-linear pattern with maximal covariation during the CIN2+ disease stage, i.e. a stage prior to invasive cancer. By using DNAm data from independent CIN2+ samples and other disease stages we further validated this result, thus demonstrating the biological and statistical significance of the non-linear dynamics exhibited by this specific CpG module. The non-linear dynamics of the module's covariation strength is reminiscent of a phase transition model, suggesting that the CpGs making up this module may be of particular biological significance. Confirming this, the DNB module exhibited a very strong overlap with 140 risk CpG sites which we identified previously using independent samples prior to neoplastic transformation [13]. We stress again that this overlap was highly statistically significant and non-trivial given that the two sets of CpGs were derived from entirely different data sets. This strong overlap meant that the DNB could predict the prospective risk of CIN2+ in cytologically normal cells 3 years in advance of morphological transformation with a statistically significant AUC value of 0.62 (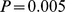). Confirming the role of the DNB CpGs as risk indicators of prospective CIN2+ status, we found that they were strongly associated with HPV status in cells of normal cytology. Moreover, the same CpGs were also associated with HPV status in head & neck cancer. Importantly, the other modules which the algorithm inferred did not exhibit maximal covariation prior to cancer, and these modules were on the whole also less interesting, exhibiting significantly lower overlaps with the previously identified 140 risk CpG sites.

In order to understand the biological significance of these findings, we generated heatmaps of the DNAm patterns of the DNB CpGs across all disease stages. This showed that these particular CpG sites exhibited a striking bi-modality, specifically within the CIN2+ stage, with some CIN2+ samples exhibiting hypermethylation at most of these sites, whilst other CIN2+ samples showed hypermethylation at only far fewer CpGs. This bi-modality is therefore a major driver of the increase in DNAm covariation at these CpG sites. In the invasive cancer stage, all of these CpG sites become hypermethylated in effectively all cancers, thus lowering the covariation. Biologically, this suggests that the emergence of an invasive cancer requires most of these CpG sites to become methylated. The observed bi-modality in the CIN2+ stage further suggests that some CIN2+ samples are much closer to the invasive cancer stage than others. This indicates that CIN2+ is a stage where epigenetic diversity across samples is maximal or near-maximal, meaning that a sample's epigenetic profile is least predictable (i.e. most stochastic). It is entirely plausible that this maximum in inter-sample epigenetic diversity reflects a disease stage where epigenetic mosaicism is also highest within individual tissue samples, consistent with the hypothesis put forward by Feinberg and colleagues that epigenetic diversity is a driver of the carcinogenic process [2]. Indeed, since the DNAm measurements considered here were taken in samples that consist of whole cell populations, a likely interpretation of the observed phase transition dynamics is a concomitant increase in epigenetic plasticity within individual samples, i.e. an increase in the number of distinct epigenetic subclones [11], [24]. This increase in epigenetic mosaicism then leads to a substantial increase in the risk of a cancer cell emerging. The subsequent decrease in variability and co-variability observed in invasive and highly proliferative cervical cancer (**Fig. 4**) may reflect either the emergence of a dominant tumour subclone or evolutionary convergence of tumour subclones. Thus, the heterogeneity or diversity in DNA methylation patterns undergoes an abrupt increase at the onset of neoplasia with more homogeneous profiles before and after this epigenetic switching point. Importantly, the variability and heterogeneity in DNAm profiles remains higher in cancer compared to normal cells (**Fig. 4**), consistent with previous observations [12].

The non-linear phase transition dynamics behaviour of the DNB module could have deep practical implications. In fact, given that the DNB was identified from only 3 stages in carcinogenesis (normal cells, CIN2+ and invasive cancer), this raises the exciting possibility that risk biomarkers could be identified from multi-stage non-prospective data, although validation will require prospective samples, as done here.

It is also important to discuss the potential merits of the phase transition DNB framework for risk prediction, in comparison to the EVORA (Epigenetic Variable Outliers for Risk prediction Analysis) algorithm [13], specially since both are able to predict prospective CIN2+ status with similar AUC values. First of all, EVORA is a univariate feature selection method that aims to identify methylation outliers, in contrast to the DNB framework which performs feature selection in a multivariate fashion relying heavily on the dynamic changes in DNA methylation covariation patterns. Hence, the DNB formalism does not rely on the existence of methylation outliers. This is an important point, because as shown by us previously, methylation outliers may not be as prominent in other more heterogeneous tissue types such as blood [17], whence why EVORA may not be applicable to prospective studies conducted in blood tissue or in surrogate tissues unrelated to the cell of origin of the cancer. Thus, the DNB framework may provide a more general mathematical framework for identifying risk biomarkers from epigenetic profiles in a wider range of tissues. Secondly, if DNA methylation changes are highly variable and dynamic in cell populations predisposed to neoplasia, then methylation levels per se may be less useful as risk biomarkers. Consistent with this, differential methylation statistics were less sensitive to identify risk markers in the prospective setting [13], [17]. Instead, the strong correlations between highly dynamic markers would provide a more stable, network-based, risk biomarker [18]. Thirdly, we have seen that the DNB formalism can identify disease risk biomarkers from non-prospective multi-stage data. Indeed, the DNB module was identified by comparing normal to CIN2+ cells and by further observing the dramatic increase in the DNB score between normal and CIN2+ cells and the subsequent drop in invasive cancer. In contrast, EVORA appears to be less useful to identify risk biomarkers from disease stages where cells have already undergone neoplastic transformation [17].

Finally, it is also important to discuss the observations made here in the context of the original dynamic system-omic model of Chen et al [18]. Even though the DNB algorithm can be motivated purely on biological grounds, without the need to invoke large dynamic changes in DNAm, the original algorithm was derived from an underlying dynamic model which assumes that DNA methylation levels may be highly variable on time-scales relevant to disease progression. Although at the single cell level, DNA methylation is a relatively stable and mitotically heritable mark, in rapidly proliferating cells DNAm replication errors can occur [25]. Most importantly, at the population level of thousands of cells, recent work has shown that DNA methylation can indeed be highly variable in time [26]. In fact, DNAm polymorphisms and stochastic variation has been observed in time course in-vitro studies of relatively large normal cell populations [26]. It is therefore plausible that in cells which are predisposed to neoplastic transformation, that DNA methylation changes are less stable, particularly, when measured across a population of cells, since specific subclones may outcompete others for a period of time, with other subclones characterised by a different DNAm profile taking over at a later stage. Supporting the view that DNAm levels can be dynamic in carcinogenic cells, we recently observed such dynamic changes in a time course Illumina 450k DNAm profiling experiment covering 480,000 CpG sites in glioma and normal neural stem cells, both treated at baseline with a BMP (bone morphogenetic protein) differentiation inducing factor (*BMP4*), and followed up for a maximum of 64 days, with measurements taken at baseline, 8, 16, 32, 48 and 64 days. Specifically, we identified a non-negligible fraction of CpGs that acquired significant hypermethylation (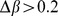) during the time course, but which subsequently lost methylation (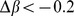) at a later time point, independently of passage number, and with more pronounced changes observed in one glioma stem cell line (**Fig. S6 in Text S1**). Thus, these data show that a proportion of DNA methylation changes in a cell population are not retained and are thus variable in time. Hence, it is equally plausible that some of the DNAm changes occuring in preneoplastic and neoplastic tissues could have a dynamic component which could indicate a particular phenotypic state of the underlying tissue.

In summary, our results are consistent with a model in which the variability and co-variability in DNA methylation increases significantly as cells approach a “switching point” between normal cytology and neoplasia. We propose that the “system-omics” DNB framework presented here should be explored further as a means of identifying disease risk biomarkers from multi-stage DNA methylation data, or from fully prospective studies profiling samples from easily accessible tissues such as blood or buccal cells collected years in advance of diagnosis. In these studies the time of sampling to disease diagnosis is variable between individuals. Hence, with sufficient numbers of individuals, the framework presented here could be exploited to identify epigenetically changing modules which could provide more robust markers of disease risk.

## Materials and Methods

### Data

The main DNA methylation data sets used in this work were all generated with Illumina Infinium 27k beadchips, have all been published previously [13], [17] and are publicly available from GEO (www.ncbi.nlm.nih.gov/geo/). See references for GEO accession numbers. The distribution of samples in each data set according to disease stage is provided in Figure 2.

### Brief review of the Dynamical Network Biomarker (DNB) algorithm

The original DNB algorithm is grounded on a dynamic “system-omic” model as described in detail in [18]. Although there the application was to gene expression data, here we adapt the formalism to the epigenetics context, and specifically to DNA methylation data. Thus, we assume that we have DNA methylation profiles across tens of thousands of CpGs and for a number of samples representing different progressive disease stages. Although the original theoretical model describes dynamic changes in time, happening in one individual, we shall consider a surrogate approach (see next subsection for justification) in which time is replaced by disease stage. Specifically, we assume that there are a number of progressive disease stages, labeled by 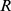, and we assume that there are 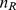 independent samples in disease stage 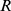. Furthermore, we assume that the transitions between specific disease stages, which we here call “switching points”, can be modelled as fold-bifurcations [18]. A theorem in dynamical systems theory, as applied to omic data, then states that a module of CpGs exists satisfying the following properties as the switching point is approached [18]:

- The variance in methylation of the module CpGs increases.
- The correlation in methylation between the module CpGs increases.
- The correlation between module CpGs and other measured CpGs decreases.

We stress that these are theoretical predictions which follow from a minimal set of fairly realistic assumptions [18]. Thus, for a given set/module 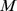 of CpGs in a disease stage 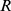, it was proposed that a score, 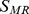 be computed as
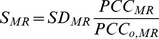
(2)where 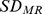 is the average standard deviation of the DNA methylation profiles of the CpGs making up the module 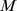 across the 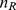 samples in disease stage 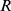, 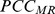 denotes their average pairwise Pearson correlation coefficient as estimated across the 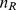 samples, and 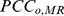 denotes the average Pearson correlation between the modules CpGs and their complement, i.e. all other CpGs not in the module 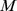. Thus, the score 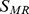 becomes maximal as the switching point is approached and would drop in value beyond this point.

It is important to note that, of course, not all CpG modules one may define or construct would exhibit an increase in this score as a switching point is approached. However, those modules which do show dramatic increases in the score between two successive disease stages are of particular interest, since the associated CpGs could then be used as disease risk indicators. These modules are called Dynamical Network Biomarkers, and hence refered to as “DNB modules” [18].

### Dynamic DNA methylation simulation model

Since acquiring dynamical genomic data from single patients is at present impractical, we propose to explore correlations in molecular profiles cross-sectionally, i.e. by taking single time point measurements (“snapshots”) of a sufficient number of different patients, all with the same stage of disease. To demonstrate that this cross-sectional approach is feasible and that computation of correlations over snapshot samples can capture dynamical network biomarkers (DNBs), we devised a simulation model. Without loss of generality, we let the simulation represent a scaled-down version of real data. Thus, we shall assume that we can measure the level of some molecular entity (here DNA methylation of a CpG site) at 500 sites (“CpGs”) in a genome, and for each of 25 patients. We shall further assume unobserved time-course molecular profiles for each of these patients (with 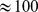 equidistant time points). Each patient is assumed to be in the same pre-disease stage, so that as time goes on, each patient approaches the critical transition point, characterised by the onset of disease. According to the theoretical framework in [18], a DNB marker exists for each of the patients and we assume that this DNB could in principle be detected by measuring the molecular profiles. The DNB itself consists of a set of highly correlated (or anticorrelated) CpGs each of which exhibits an increase in methylation variance as time approaches the critical point. At earlier time points the correlation of the DNB genes is either absent or is masked by other sources of variation. The precise DNB is allowed to vary from patient to patient, but we also allow for some overlap. This is biologically justified since we know that particular genes are more likely to be targets for differential methylation or differential expression in early stage disease. For instance, the importance in cancer of PolyComb Group Target genes (PCGTs), which account roughly for over 1500 genes in the human genome is undisputed [4], [5] and further evidence for their role in preneoplastic disease was presented in [13]. Thus, we assume that the specific DNBs in each patient are drawn from a common pool of 50 “risk-PCGTs”, i.e 10% of the 500 features, which is roughly the proportion of PCGT genes to all genes in the human genome. However, we want the patient-specific DNBs to vary from patient to patient, so the CpG (or gene) set for each patient-specific DNB is constructed by randomly sampling 20 of the 50 CpGs. Thus, the expected overlap of any two patient-specific DNBs is small and on the order of 8 CpGs. This again is similar to the inter-sample overlap of risk-CpGs observed in [13]. For each of the 25 patients we thus construct a hypothetical time course of molecular profiles over 500 CpGs and 100 time points. For the 20 DNB CpGs selected from the pool of 50 risk PCGT CpGs, we take their methylation values to vary in time according to
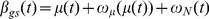
(3)where 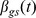 denotes the methylation 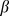 -value at CpG site 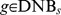 in individual 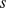 at time 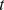 (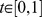), *averaged over a large number of cells making up the sample*. In other words, the simulation model is formulated not at the single cell level, but at the cellular population level. We further note that the methylation 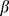 -value at CpG 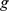 in sample 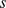 represents the fraction of cells that have that particular CpG site methylated. Thus, this value is bounded between 0 and 1, as one would expect for a random variable from a beta distribution. Formulating the simulation model on this scale has the advantage that the values are readily interpretable as methylation fractions. In the above model, 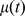 describes the increase in the mean methylation level with time and we assume this takes the form
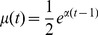
(4)with a parameter 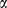, which we choose to be 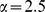. The exact value of this parameter is not important, but we are implicitly assuming (without loss of generality) that the CpG sites start out unmethylated with 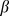 -values around 0.05. The maximum attainable mean value is 0.5 corresponding to 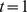. This assumption too is not necessary but is convenient as explained below. We point out that the non-linear increase in mean methylation is also not essential and we could replace the above exponential with a linear function of time. The second term in equation 3 describes the increased variability in methylation as a function of the mean methylation, whereas the third term represents an intrinsic biological and technical noise which we shall assume to be stationary so that 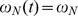.

Since, as explained above, methylation data is beta-distributed we must reformulate the above in the logit-transformed basis
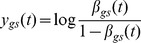
(5)in order to correctly incorporate the noise term which is Gaussian in the logit basis, i.e.
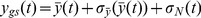
(6)where 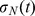 denotes random normal deviates. Specifically, we assume that [27]

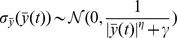


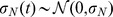
where 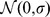 denotes a normal deviate of mean zero and standard deviation 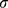, and 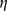 and 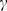 are parameters that we take to have values 1 and 0.8, respectively. These values are chosen to generate realistic profiles. To justify the above model for the variance, we first note that the DNA methylation measurements are taken over large collections of cells. Thus, when the mean methylation is 0.5, half of the cells have the particular CpG site methylated (assuming a binary methylation state), while the other half have this site unmethylated. Thus, at this point the expected variability in methylation across the cell population is maximal. With the above model, the variance in methylation is indeed maximal at 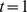 since 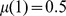 and 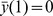. The above model also ensures that the rate of variance increase grows as the transition point is reached in line with previous models [27]. The constant value 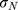 is chosen to be equal to 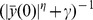, as required, since it should equal the intrinsic stochastic variation at the initial time point.

For the 450 non-PCGT CpG sites we assume an uncorrelated stationary pattern, i.e.
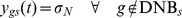
(7)and so these features remain stably unmethylated.

Finally, to generate the methylation profiles on the appropriate scale, we transform the 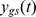 values back into 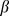 -values using the inverse of equation 5. We note again that since variability in DNA methylation patterns is maximal at 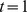, we consider 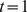 the transition or switching point representing the onset of a new disease stage, e.g. neoplasia.

Having generated hypothetical time course molecular profiles 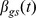, we next sample for each individual 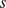 a random time point 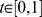, thus defining a 500×25 “static snapshot” matrix 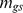. This data matrix represents what in practice would be the observed data.

### Identification of the DNB in the simulated data

Given this observed data matrix, the question is now whether clustering over the genes can identify a module of CpGs which captures a “consensus” DNB across all the considered patients. This consensus DNB should consist of the selected 50 risk “PCGT” CpG sites. To test this we first compute from 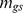, the corresponding correlation matrix between the CpGs. Next, from this correlation matrix we construct an unweighted adjacency matrix with edges representing significant pairwise correlations, and finally use the spectral decomposition algorithm [28] to identify modules (i.e. communities of relative large edge density) in the resulting graph. Next, we select the module with the largest variance in methylation (here we average the standard deviation in methylation of the constituent module CpGs) across samples and declared this to be the candidate DNB. Sensitivity and positive predictive values (PPV) are then calculated and significance P-values of overlap between the true 50 DNB markers (i.e the 50 risk PCGT CpGs) and those found in the candidate DNB are estimated using a binomial test. A total of 100 different Monte Carlo runs (in each run new time-course DNA methylation profiles and a new observed data matrix is generated) were performed to test for robustness.

### Construction of candidate DNB modules in cervical carcinogenesis

Candidate DNB modules were inferred by adapting a previous procedure [18]. Briefly, we compared DNA methylation profiles between the normal and CIN2+ samples of set LBC1 and the normal and cervical cancer samples of set CC (see **Fig. 2**). Specifically, we used a Bayesian shrinkage linear model (limma) [29] to derive in each case a ranked list of top discriminative CpGs. The list of CpGs was selected using a false discovery rate cutoff of 0.05 and imposing a threshold on the standard deviation in DNAm as measured across the CIN2+ or cancer samples, respectively. The thresholds on the standard deviations were chosen from density plots of the standard deviation across all CpGs, which revealed clear boundaries where the density dropped significantly. The resulting thresholds were in the range 0.05–0.1. However, in cases where too many CpGs passed this threshold, we capped the number of CpGs at a maximum of around 1000. To identify clusters/modules we then applied the robust partitioning around medoids algorithm [30] with a Pearson distance correlation metric and with the number of clusters variable between 2 and 10. The optimal number of clusters was estimated using the average silhouette width [30] and was found to be two in both the LBC1 and CC data sets, resulting in 4 modules in total, denoted as (LBC1-A, LBC1-B, CC-A, CC-B).

### Module score computation and identification of the DNB in cervical carcinogenesis

For each of the four inferred modules, a module score was first estimated in the two training data sets, i.e. LBC1 and CC (see **Fig. 2**), according to the equation 2 above [18]. Briefly, the estimation of the score also requires a normal reference. For each disease stage we used as normal reference the phenotypically normal specimens of the corresponding study profiling the samples. For the CIN2+ (all HPV+) samples from LBC1 we used the normal samples of that same study as reference. For the cervical cancer samples we used as reference the 15 normal tissue samples from the same study. Thus, for each CpG in a module we first estimated the mean and standard deviation in DNAm across the normal reference samples. The DNAm profiles across the samples in the disease stage where then renormalised relative to this reference, resulting in normalised z-scores reflecting deviations from the normal reference. The standard deviation of these z-scores across the samples in the disease stage were then computed for each CpG in the module. The average of these standard deviations defines the measure 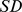 in equation 2. The average of the absolute pairwise Pearson correlations between the CpG DNAm profiles in the module defines the measure 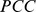. To estimate the correlations of the module CpGs to other CpGs not in the module, we randomly selected CpGs in the complement, computing the absolute correlations of the module CpGs with these and then averaging. A global average was obtained by running this randomisation procedure a total of 10 times, which resulted in stable values. Finally, the score for a candidate DNB module was computed as in equation 2.

To identify DNBs we then studied the profile of the scores for each of the 4 modules (LBC1-A, LBC1-B, CC-A, CC-B) across the 3 main stages represented in the LBC1 and CC datasets, i.e. normal cells, CIN2+ and invasive cervical cancer. A DNB is a module exhibiting a characteristic dramatic increase in the score (at CIN2+ stage) with a subsequent drop at a later stage (i.e. invasive cancer).

### Testing of the modules scores in independent data

In order to test reproducibility, the module scores of the four modules were also estimated in an independent data set (LBC2) consisting of normal cells and CIN2+ samples. Thus, this allowed the reproducibility of the score values obtaining in the CIN2+ disease stage of dataset LBC1 to be assessed in a completely independent set (LBC2). In addition, module scores were also evaluated in another independent dataset (ART), profiling normal HPV−, normal HPV+ and precursor CIN2+ cells (both HPV− and HPV+). Because the latter three stages are intermediate between normal HPV− and CIN2+, we can further validate the framework since the prediction is that the values for the intermediate stages should also be intermediate between those for the normal HPV− and CIN2+ stages. We note that as normal reference in the score computation we used the normal samples of LBC2 for the CIN2+ stage of LBC2, and the normal HPV− samples of ART for the other 3 stages of the ART set. We remark that in the ART set we had many more samples, allowing the normal HPV− state to be considered separate from the normal HPV+ state. However, results are unchanged if they had been merged.

## Supporting Information

Text S1 The additional file/supplementary information document contains Supplementary Figures S1, S2, S3, S4, S5, S6 plus their legends/captions.
(PDF)
