# Supplementary material for: The Dynamics of DNA Methylation Covariation Patterns in Carcinogenesis
Source: PLoS Comput Biol. 2014 Jul 10;10(7):e1003709. doi: 10.1371/journal.pcbi.1003709 (PMC4091688; doi:10.1371/journal.pcbi.1003709)
Supplement: Text S1 — The additional file/supplementary information document contains Supplementary Figures S1, S2, S3, S4, S5, S6 plus their legends/captions. (PDF) [file pcbi.1003709.s001.pdf]

Additional File/Supplementary Information for manuscript

“The dynamics of DNA methylation covariation patterns in carcinogenesis”

Andrew E. Teschendorff *, Xiaoping Liu, Helena Caren, Steve M. Pollard, Stephan Beck, Martin Widschwendter and Luonan Chen.

*Corresponding Author: andrew@picb.ac.cn, a.teschendorff@ucl.ac.uk

SUPPLEMENTARY FIGURES:

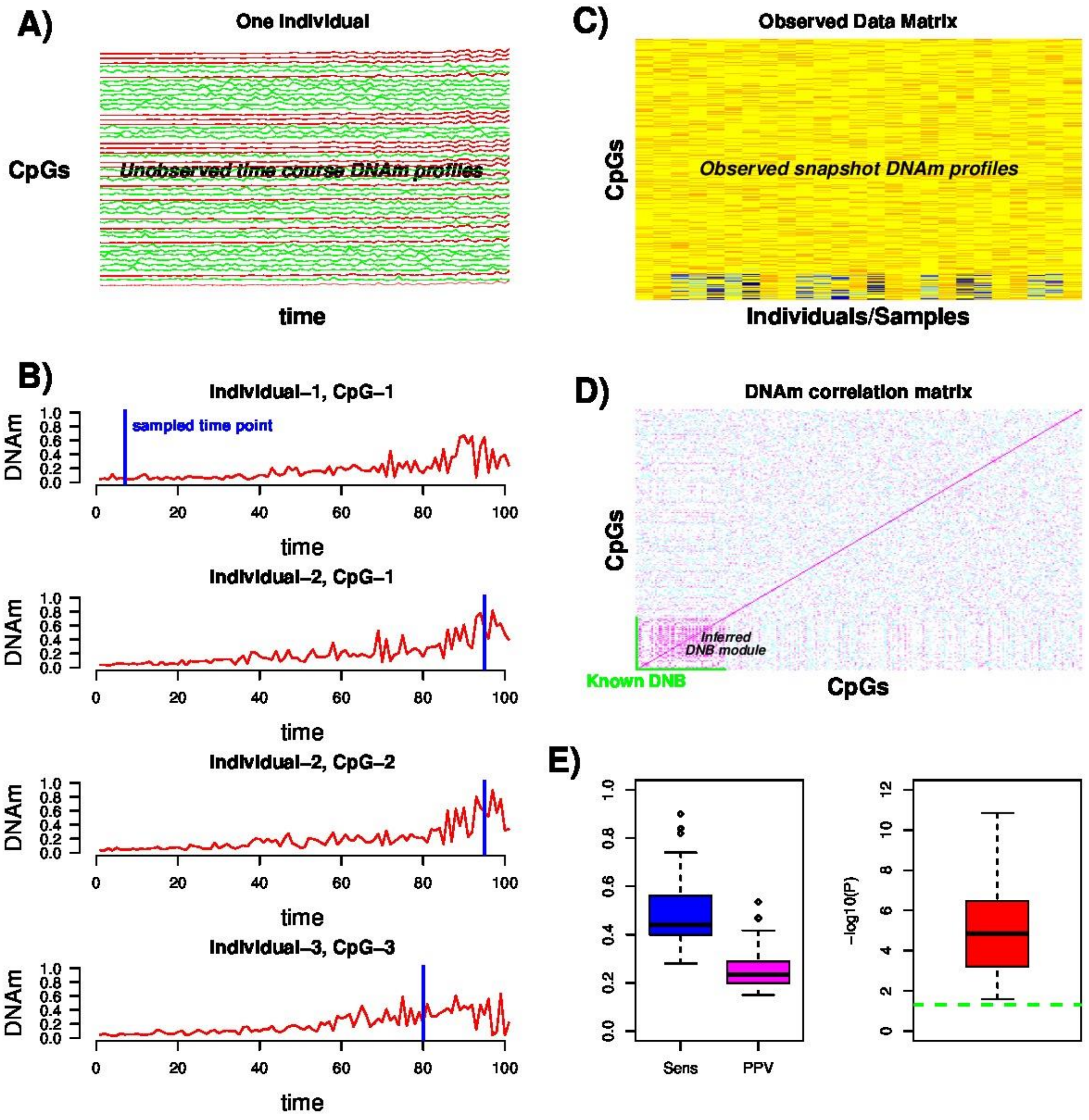

Fig.S1: A) Simulated examples of CpG methylation profiles changing with time (unrecorded data) in one individual. Red indicates CpGs where the mean and variance in methylation increases with time, green indicates loci where methylation changes are stable. **B)** Zoomed in version of A) for specific CpGs undergoing stochastic increases in methylation with time. We depict such CpG DNA methylation (DNAm) profiles for a common CpG in two individuals and different CpGs in the same individual as well as distinct CpGs in different individuals. Observe how the variance increases as time approaches the critical transition point (e.g. onset of neoplasia). The vertical lines indicate the randomly sampled time points at which DNAm is measured for each individual, from which the observed data matrix is then constructed. **C)** Heatmap of the observed DNA methylation data matrix, obtained by sampling one time point from each individual in B). Note how the first 50 CpGs (the DNB markers) show methylation variability and that samples have not been time-ordered since for individuals in the same pre-disease stage the exact timepoint in relation to diagnosis is generally unknown. **D)** Heatmap of DNA methylation correlations (pink=high,cyan=low) identifies a highly correlated gene module, the Dynamical Network Biomarker (DNB), which agrees well with the known DNB markers (indicated in green). **E)** Simulation results assessing how the inferred DNB (see Methods) overlaps with the known DNB. Left panel shows the sensitivity and positive predictive

values across 100 Monte Carlo runs. Right panel shows the significance (-$\log_{10}$ P) across the 100 runs. Green dashed-line represents significance at the P=0.05 level.

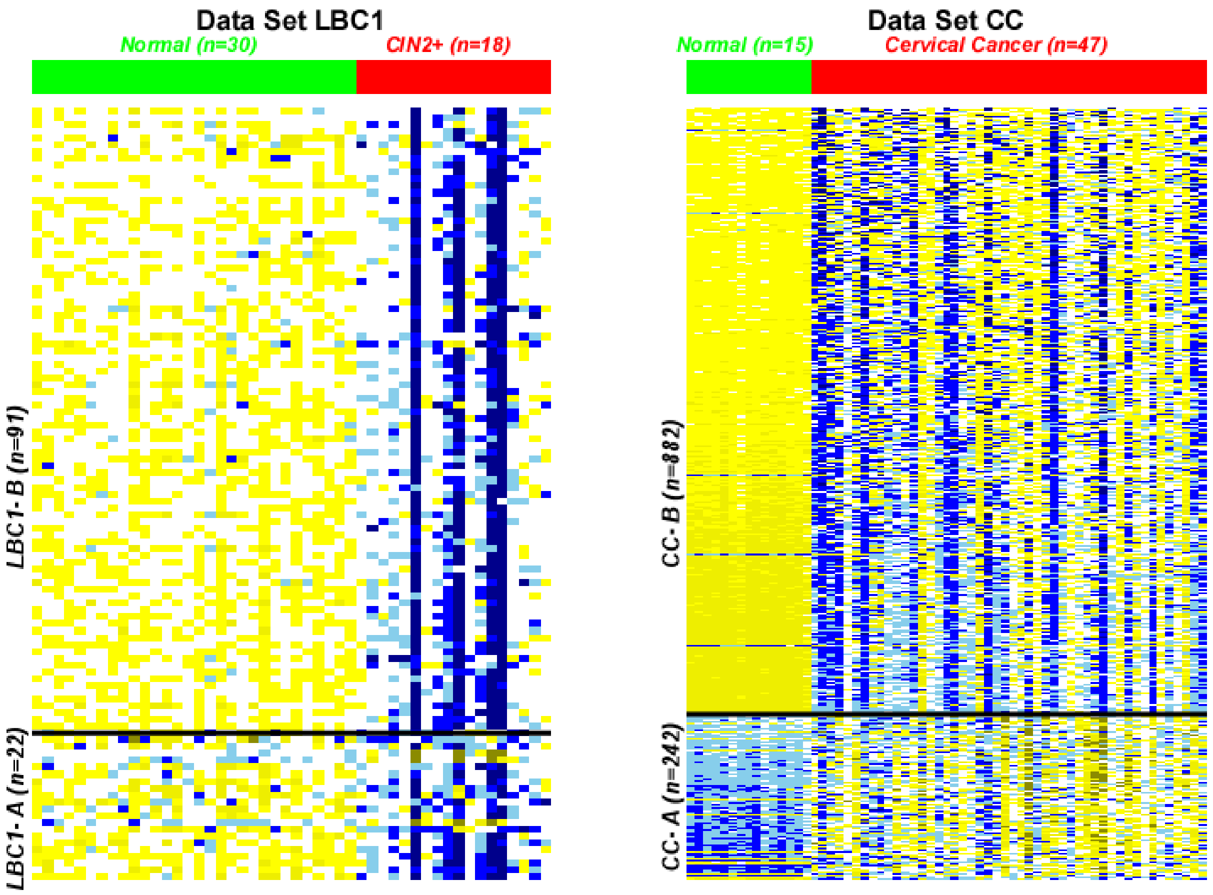

Fig.S2: Heatmaps of the CpG modules (LBC1-A, LBC1-B, CC-A, CC-B) inferred via the DNB algorithm from data sets LBC1 and CC, as indicated. Samples have been ordered according to histology, as indicated. The number of CpGs in each module is as indicated. Methylation beta-values have been standardised to mean zero and unit variance to help visualize the differences in methylation between the samples of different histology. Yellow colour indicates relative low methylation, darker tones of blue indicate correspondingly larger methylation values. The black horizontal lines delimit the two CpG modules in each data set.

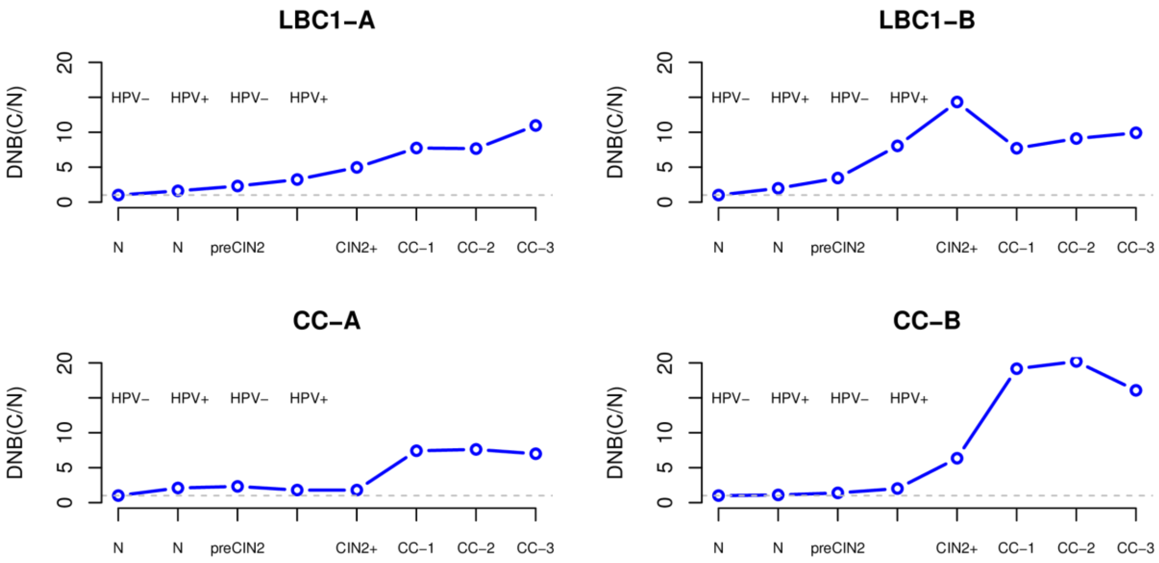

Fig.S3: The dynamic changes in the module relevance score (indicated on the y-axis as DNB(C/N)) of the four inferred modules, as a function of disease stage: the stages shown are N(HPV-), N(HPV+), preCIN2(HPV-), preCIN2(HPV+), CIN2+, CC stages 1,2 and 3. (Abbrev: N=Normal, preCIN2+: precursor CIN2+ cells, CIN2+=cervical intraepithelial neoplasia of grade 2 or higher, CC=cervical cancer).

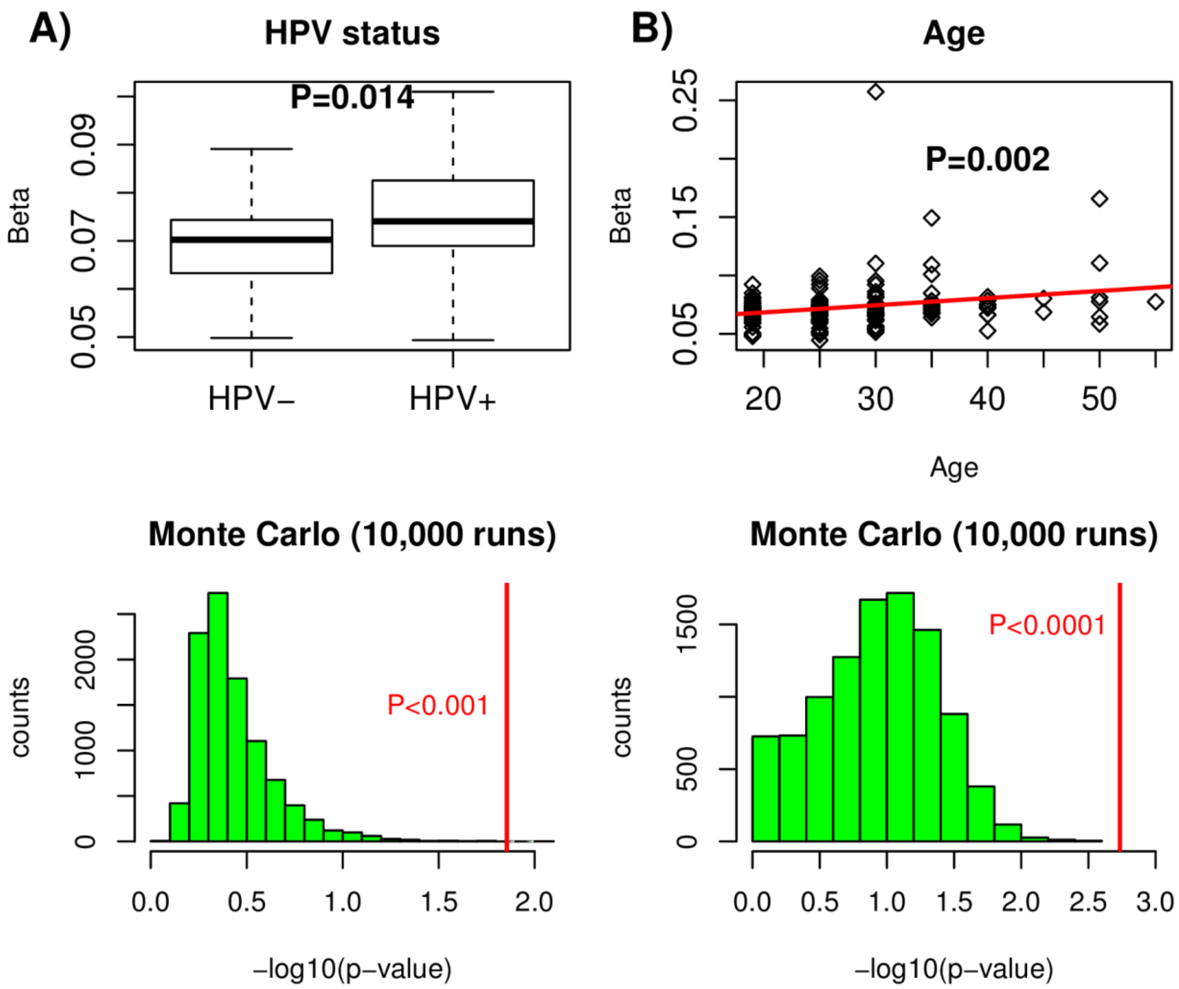

Fig.S4: A) Upper panel: association of the average methylation of the 91 DNB (LBC1-B) CpG markers with HPV status in cytologically normal CIN2+ precurors cells. P-value from a Wilcoxon rank sum test is given. Lower panel: Monte Carlo analysis in which a random set of 91 CpGs were selected and P-value recomputed (10,000 runs). Red line indicates the observed –log10(p-value), green histogram is the one expected by random chance. **B)** As A) but for age. In the case of age, the linear regressions were adjusted for prospective CIN2+ status and HPV status. All samples in this figure were taken from the ARTISTIC cohort.

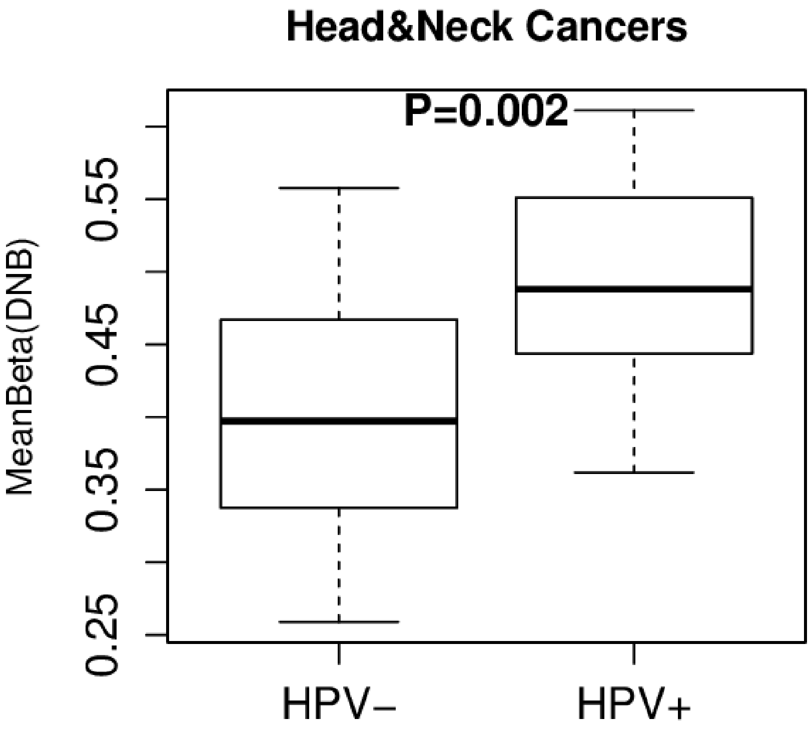

Fig.S5: Association of the average methylation of the 91 DNB (LBC1-B) CpG markers with HPV status in the Head & Neck Cancer set of Lechner M et al Genome Med. 2013. P-value from a Wilcoxon rank sum test is given.

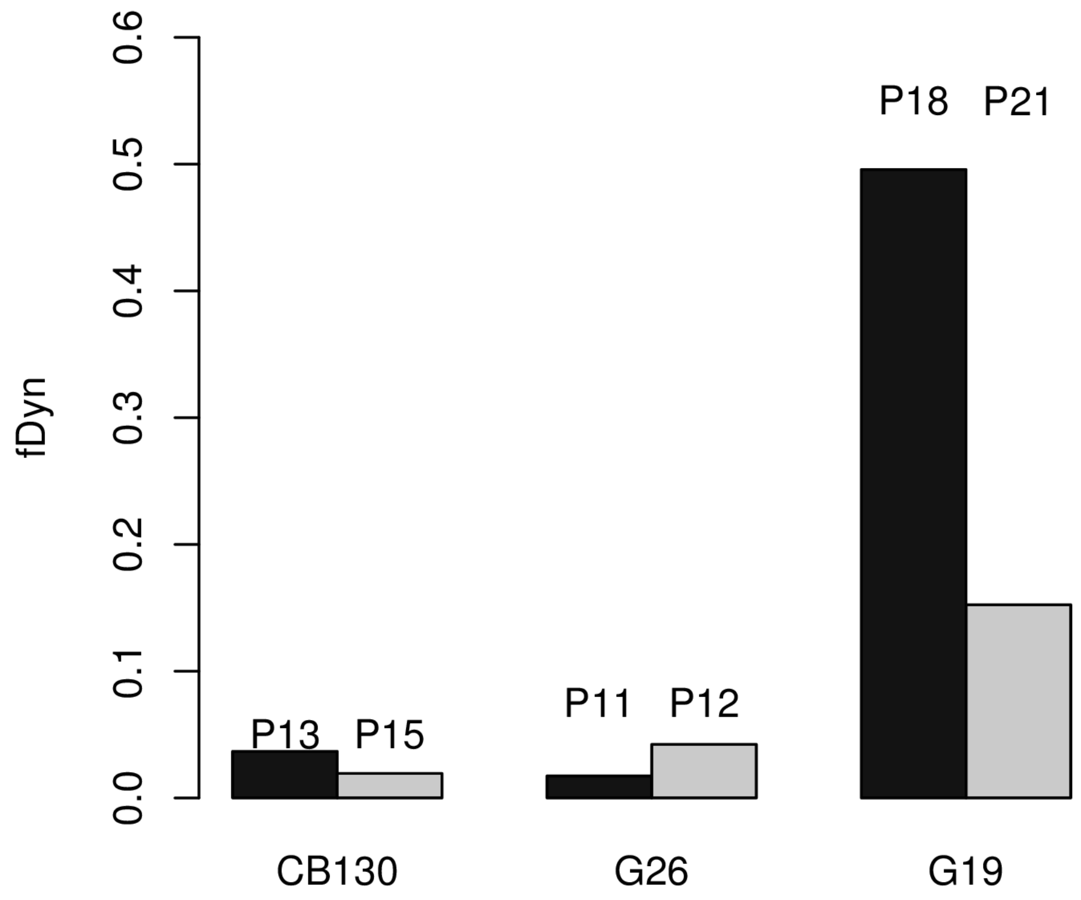

Fig.S6: For a normal neural stem cell (CB130) and two glioma stem cells (G26 \& G19) we show on the y-axis (fDyn) the fraction of CpGs exhibiting significant increases ($\Delta\beta$ > 0.2) and subsequent decreases ($\Delta\beta$ < -0.2) in DNA methylation in a time course experiment following induction with the BMP4 differentiation factor. For each cell line, results are shown for two different passage numbers (P13 and P15 for CB130, P11 and P12 for G26, and P18 and P21 for G19). For instance, for the glioma stem cell line G19, more than 15% of CpGs that showed a significant increase in DNA methylation at any point during the time course, subsequently also showed a significant decrease before the end of the time course was reached. DNA methylation was measured with the Illumina 450k platform and samples taken at baseline (BMP4 induction), and at 8, 16, 32, 48 and 64 days after BMP4 induction.
